# Enhanced leachate phytodetoxification test combined with plants and rhizobacteria bioaugmentation

**DOI:** 10.1016/j.heliyon.2023.e12921

**Published:** 2023-01-13

**Authors:** Isni Arliyani, Bieby Voijant Tangahu, Sarwoko Mangkoedihardjo, Enny Zulaika, Setyo Budi Kurniawan

**Affiliations:** a Department of Environmental Engineering, Institut Teknologi Sepuluh Nopember (ITS), Surabaya, 60111, Indonesia; b Department of Biology, Institut Teknologi Sepuluh Nopember (ITS), Surabaya, 60111, Indonesia; c Department of Chemical and Process Engineering, Faculty of Engineering and Built Environment, Universiti Kebangsaan Malaysia, 43600 UKM Bangi, Selangor, Malaysia

**Keywords:** *Cyperus papyrus*, *Nitrosomonas communis*, *Bacillus cereus*, *Pseudomonas aeruginosa*, *Typha angustifolia*

## Abstract

Plant combination and rhizobacterial bioaugmentation are the modification of constructed wetlands (CWs) to promote the detoxification of leachate. In this study, characterization of leachate was carried out to ensure the maximum concentration of leachate that did not affect the plant's growth. Herein, the identification of leachate-resistant rhizobacteria is used to determine the type of bacteria that is resistant and has the potential for leachate processing in the next step. The phytodetoxification test is carried out by comparing the addition of rhizobacteria and without the addition of rhizobacteria to detox leachate parameter Chemical Oxygen Demand (COD), Biological Oxygen Demand (BOD), Total Suspended Solid (TSS), Total Nitrogen (TN), Cadmium (Cd), and Mercury (Hg). Results showed that used plants could still live in the largest leachate concentration of 100%. The rhizobacteria that were identified and bioaugmented in the reactor were *Bacillus cereus*, *Nitrosomonas communis*, and *Pseudomonas aeruginosa*. Phytodetoxification test by a single plant showed the efficiency ranged between 40% and 70%. The addition of rhizobacterial bioaugmentation and plant combination can improve the percentage of COD 80.47%, BOD 84.05%, TSS 80.05%, TN 75.58%, Cd 99.96%, and Hg 90%. These modifications are very influential for leachate detoxification through plant uptake and rhizodegradation processes.

## 1 Introduction

Several environmental problems are caused by rapid urbanization and population growth, one of which is municipal waste management, which is getting greater with only landfilling applied [47]. The waste undergoes a series of physicochemical and biological transformations after being dumped, thus producing leachate that can contaminate surrounding soil, groundwater, and surface water [141]. Several physicochemical parameters affect the quality of leachate landfill. Furthermore, those parameters are pH, suspended solids (SSs), biological oxygen demand (BOD), chemical oxygen demand (COD), ammonia (NH_4_–N), total nitrogen (TN), chloride, phosphorus, heavy metals, and alkalinity [26,71]. Therefore, the solution to handling leachate can be employing phytotechnology by using plants as leachate processing agents, called phytotreatment or phytoremediation for leachate that has polluted waters and/or soil [69,101].

In addition, it is necessary to conduct testing before applying phytotreatment or phytoremediation to determine the effect of contaminants on plants using phytotoxicity tests [155] The plants *Scirpus grossus* and *Cyperus rotundus* used in the tempeh industrial wastewater treatment can be accepted for about 25% of the waste content [105]. Various types of plants are used in phytotoxicity, such as *Phragmites australis*, *Acorus calamus* [27], *Chrysopogon zizanoides* [43]. [135] using *Sinapsis alba* L. and *Lemna minor* L. as bioindicators to measure the toxicity of leachate. However, it remains unknown whether or not the maximum ability of plants to tolerate leachate is where the leachate content varies in different places, hence, a toxicity test is necessary [15]. Phytotoxicity testing contains several parameters that can be analyzed: fresh weight, changes in the shoot or root length during testing, or physical observations such as chlorosis, yellowing of leaves, and cupping of leaves [30,110]. The Range finding test is one of the steps that must be done before conducting the phytotoxicity test. This is a preliminary test to determine the concentration of contaminants at which plant species can survive in leachate-contaminated media or leachate treatment as the first step of a phytotoxicity study [7,8,28].

*C. papyrus* and *T. angustifolia* are plants in wetland areas such as swamps, riverbanks, and roads with various benefits [68,82,97,117,131]. Meanwhile, the plants *T. angustifolia* and *C. papyrus* can well adapt to surface water and free subsurface water flows [11,72,[147], [164]]. *T. angustifolia* can survive in low pH environments and anaerobic conditions. Moreover, *T. angustifolia* grows through rhizomes and needs a lot of sunlight to survive. These plants can treat wastewater, leachate, and remediation [25]. *C. papyrus* and *T. angustifolia* can remove pollutants 60% higher than without plants in the wetland [160].

Besides the presence of plants, degradation by microorganisms in the process of removing additional pollutants is also an essential part of phytotreatment [3,6]. These microorganisms have significant contributions to plants' growth and physiology. In soils with high heavy metal content, these rhizosphere bacterial populations play important beneficial roles in plants’ responses to pollutants [52,116]. Plant growth-promoting rhizobacteria (PGPR) can increase plant growth through various mechanisms, including phosphate dissolution, biological nitrogen fixation, and so on [78]. The *Simplicispira* genus can generally denitrify NO_3_^−^N and NO_2_^−^N under aerobic and anaerobic conditions. However, its presence is abundant in phytotreatment with plants (7.1%), meanwhile, the relative abundance is only 1.7% [100]. In addition, common genera that can perform denitrification include *Thauera* (5.6%), *Denitratisoma* (5.0%), *Thiobacillus* (4.1%), *Pseudomonas* (3.7%), *Longilinea* (3.3%), *Zoogloea* (1.5%), and *Desulfovibrio* (1.4%) [125]. When heterotrophic denitrification occurs, *Pseudomonas* and *Azoarcus* take major roles in contributing to COD and N removal. Furthermore, nitrogen-converting microorganisms including *Nitrosomonas*, *Nitrosospira*, *Zobellella denitrificans*, and *Pseudoxanthomonas* coexist with anaerobic microorganisms. *Rhodobacter* sp., *Dechloromonas*, *Bacillus*, and *Hyphomicrobium* are some of the microorganisms that are present in pollutants and can also be used for the growth of aerobic and anaerobic microorganisms in the media [146].

The distribution of microbial communities is closely related to removing pollutants from leachate. The relative abundance of rhizobacteria in the soil increases with pollutant concentrations and only focuses on the capabilities of leachate processing plants. Meanwhile, several studies focused on the identification of bacteria for wastewater treatment have already been conducted. In contrast, research on the resistance of leachate bacteria isolated from the rhizosphere, and the potential to improve the process has not been widely explored [67]. Especially, explored the capability of plants mediated treatment of Hg and Cd [121]. also studied the performance of six native Indian plants to treat pulp and paper wastewater which result in a high accumulation of Cr and Cd. Meanwhile, the treatment of landfill leachate by fungal species of *Aspergillus flavus* showed up to 98.81% removal of pollutants in 25% leachate concentration [162]. Research on the bioaugmentation of rhizobacterial species (*Vibrio alginolyticus*) to treat aluminum-contaminated wastewater was demonstrated by Ref. [104] with a 14% increment as compared to the control reactor (without bioaugmentation). Relatively few studies have been conducted at present on the characterization of microbial communities in plant roots resistant to landfill leachate. However, studies on the bioaugmentation of rhizobacterial species combined with phytodetoxification of landfill leachate using native species of *C. papyrus* and *T. angustifolia* is still rare.

Therefore, the current research aims to determine (i) leachate characterization in the location, (ii) the effect of combination plants on the leachate treatment, (iii) the resistance of leachate bacteria isolated from the rhizosphere of *C. papyrus* and *T. angustifolia*, and (iv) the effect of bioaugmentation on the leachate removal as a developmental strategy for the future phytodetoxification of leachate using the constructed wetland to fulfill these gaps. Thus*,* it is hypothesized to reduce pollutants better because of the symbiosis of plants and rhizobacteria by understanding the potency of leachate-resistant rhizobacteria from *C. papyrus* and *T. angustifolia*. This research is limited to the initial analysis under laboratory scale with the promising result expected to contribute to the tertiary treatment for the reduction of landfill leachate toxicity before disposal into the water surface.

## 2 Material and method

### 2.1 Characterization of leachate

This research was conducted on a laboratory scale. The leachate samples were taken from the inlet of the leachate holding pond at the Griyomulyo Landfill, Jabon, Sidoarjo. The sampling location of leachate is shown in the supplementary data. In this study, the initial parameter analysis of leachate was chosen as a research variable based on the Quality Standards of the Minister of Environment Regulation No. 59 of 2016 and the USEPA International Quality Standards, namely Total Nitrogen (TN), Heavy Metals Cadmium (Cd) and Mercury (Hg), BOD, COD, TSS, and pH (USEPA, 2000). Total nitrogen (TN) and COD were analyzed using a colorimeter DR 900 (HACH, USA). Then, BOD was measured after five days of incubation at 20 °C with a DO meter DO-5510 (MRC). Subsequently, total suspended solids (TSSs) were determined using the gravimetric method, and pH was measured in situ using a portable ST300G (OHAUS). Meanwhile, for heavy metals, Cd was analyzed using Flame-AAS instrument based on SNI 6989–84:2019 standard method, and Hg analysis was based on SNI 6989–78:2019 with Cold Vapor AAS.

### 2.2 Range finding test (RFT)

#### 2.2.1 Media preparation

The media used were 1 kg of gravel in the bottom, 1 kg of sand, and 1 kg of soil. The total media was 3 kg in a 10 L volume container. The media was sieved to remove coarse fragments and get the same size [19]. Then, the container was filled with 3 L of water to keep the sand moist because the plants used are aquatic plants.

#### 2.2.2 Plant preparation

Two types of plants of *C. papyrus* were in different containers with *T. angustifolia* obtained from growth/harvest results of the same age [79,105]. Plants used in RFT were second-generation plants [106], then the plants were planted in containers and acclimatized for 1 week to ensure that plants could live and adapt.

#### 2.2.3 RFT

The leachate concentration used in this study refers to the USEPA Ecological Effect Guidelines OPPTS 850.4400. RFT was carried out with different concentrations of 0%, 10%, 25%, 50%, 75%, and 100%. The comparison of the leachate concentration used was made by diluting the leachate with the volume of tap water [[105], [166]]. Then, plants were watered with 2 L of leachate every 3 days. Physical observations were carried out daily by measuring plant height in aerial plants which was then made a growth rate graph to determine the different effects on each concentration [46]. The time of the RFT test was 4 days/96 h, however, if there was no change in conditions, it was extended by 24 h to 14 days.

The percentage (%) of wilted plants at each concentration was determined by dividing the number of wilted plants by the number of plants in the container and observations of plants' growth were carried out physically and daily [23,156]. The highest concentration was chosen wherein the plant was still alive with good conditions that did not cause withered plants and was in good condition in terms of its physical characteristics. This concentration was used for research tests conducted in wetlands. The characteristics of plants were observed by measuring the aerial plants’ height using tape measurement to make sure they remained in good condition.

### 2.3 Identification of leachate-resistant's rhizobacteria

#### 2.3.1 Sample preparation

**Fig. 1:**
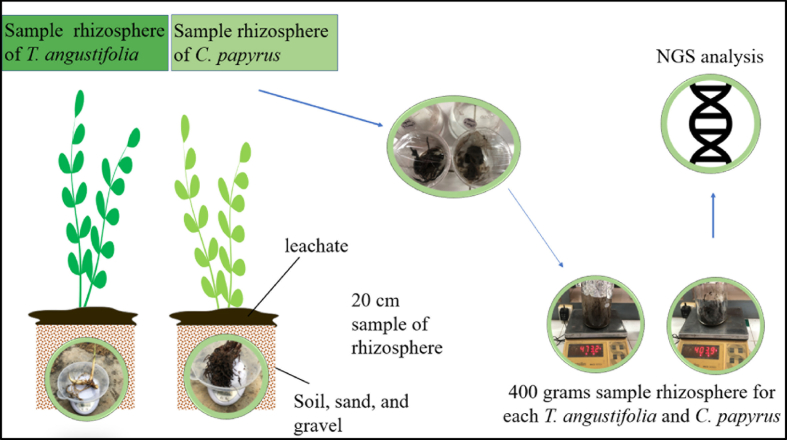
Sampling process for rhizobacteria identification analysis.

Root samples of *T. angustifolia* and *C. papyrus* were taken initially from the previous test, that is the RFT, where plants were given pure leachate for 2 weeks with different plants in different reactors, as shown in Fig. 1. Isolation of rhizobacteria was taken from the soil area around the plant roots with a depth of about 20 cm [50]. The root of the plant is taken approximately 400 g including root plant and soil. Then, the sample is directly sent to PT. Genetika Science for Next Generation Sequencing (NGS) analysis.

#### 2.3.2 Next Generation Sequencing (NGS)

Samples were analyzed at PT. Genetika Science, Banten, Indonesia with NGS-16 S Sequencing. 16 S Sequencing has a high level of taxonomic and phylogenetic resolution for rhizobacteria identification [20]. Identification of rhizobacteria with NGS starts from the genomic DNA of each sample isolated using Quick-DNA Fungal/Bacterial Kits from Zymo Research. NanoDrop spectrophotometer and Qubit fluorometer were used to determine the DNA concentration.

#### 2.3.3 Identification of rhizobacteria

Library preparation was carried out using Kits from Oxford Nanopore Technology. MinKNOW software version 20.06.9 was used for GridION sorting [144]. Moreover, the quality of FASTQ files was visualized using the Nanoplot [36]. The filtered filters were classified using the Centrifuge classifier [61]. Bacteria and Archaea indexes were downloaded from the centrifuge website (https://ccb.jhu.edu/software/centrifuge). Analysis and visualization of the phylogenetic tree were performed based on rRNA genes with Sankey using Pavian (https://github.com/fbreitwieser/pavian) and Krona Tools (https://github.com/marbl/Krona). Then, the results of the identification of rhizobacteria were discussed based on the literature review to find out the benefits of leachate processing that will be needed for further research.

### 2.4 Phytodetoxification test

A batch system was used in this research. There were three reactors needed with triplicate and one reactor control without plant and bioaugmentation [16]. The concentration used was the highest leachate concentration that can be accepted by plants from the previous method which was 100%. Following are the compositions of each reactor using soil as high as 5 cm, small gravel as high as 10 cm, and large gravel as high as 5 cm [126], meanwhile, the leachate needed to meet the needs are as high as 5 cm. The phytodetoxification test is divided into two steps.

a. Compare each plant (single plant) versus plant combination

First, *T. angustifolia* and *C. papyrus* are used in different reactors called single plant use, meanwhile, the other reactor is for both of the plants called plant combination. Each reactor was planted with six plants of *T. angustifolia* and watered with 100% leachate for 30 days and the sample was analyzed every 6 days. For the plant combination, three plants of *T. angustifolia* and three plants of *C. papyrus* were used. The same treatment was also used for *C. papyrus* and combination. The best result would be used in the subsequent step.

b. Compare the without versus with bioaugmentation

The best result from step 1 was a combination of plants used for this step to compare with the addition of rhizobacterial and without bioaugmentation. The rhizobacteria used were identified with high potential for detox pollutants which were *Pseudomonas aeruginosa*, *Bacillus cereus*, and *Nitrosomonas communis*. The three rhizobacteria in the amount of 2 (v/v) were added to the reactor. The sampling treatment is the same as in step 1.

c. Statistical Analysis

Statistical analysis of the normality test was initially performed using the Shapiro-Wilk method. The results were normally distributed based on the normality test. Afterward, an analysis of variance (ANOVA) was also performed [70]. A generalized linear ANOVA was chosen to determine the correlation between variables, and Tukey's test of real difference with = 0.05 was used to determine the significance of the results [53].

## 3 Result and discussion

### 3.1 Characteristics of landfill leachate

Except for heavy metal Cd and Hg which are below the quality standard, results of the concentration characterization on each parameter have not met the quality standard of the Minister of Environment Regulation No. 59 of 2016 concerning leachate wastewater. The leachate characteristics of the Griyomulyo landfill are shown in Table 1.

**Table 1:** Characteristics of leachate at the Griyomulyo landfill, Jabon, Sidoarjo.

Parameter	Unit	Analysis	Quality Standart	Description
pH	–	4.59	6–9	Does not meet
BOD	mg/L	1140	150	Does not meet
COD	mg/L	9216	300	Does not meet
TSS	mg/L	120	100	Does not meet
TN	mg/L	70	60	Does not meet
Hg	mg/L	0.00353	0.005	Fulfil
Cd	mg/L	0.0516	0.1	Fulfil
Reference	–	Analysis result, 2021	Minister of Environment Regulation No. 59 of 2016	

Landfill leachate values in other areas, namely Sambutan Landfill, Samarinda aged 1–5 years, have parameters above the quality standard except for TSS with a value of 45.92 mg/L [58]. In another study at the same location, the Griyo Mulyo Landfill in 2018 had COD, BOD, TSS, and TN values that did not meet the quality standard of 4120 mg/L; 3862.5 mg/L; TSS 220 mg/L; and 3488.4 mg/L [89]. Thus, following its characteristics (Table 1), it is recommended to carry out processing before discharging into the environment because it can spread pollution [81].

Landfill leachate is interpreted as a liquid resulting from the percolation of rainwater that comes seeping through solid waste in landfills, as well as water vapor present in waste products and waste degradation [32,134]. However, rainfall, evapotranspiration, groundwater infiltration, runoff, and the level of compaction in the landfill are the primary influencing factors on leachate production [85]. Therefore, various techniques are used to control water ingress into the landfill, including installing impermeable layers and covers to minimize leachate [14,34,135]. The analyzed leachate samples were taken from leachate near the landfill that was still operating to be categorized as young leachate. Further research was focused on parameters that exceed quality standards to be processed with constructed wetlands so that they were safe to return to the environment.

Leachate will be applied in the range of BOD 1500–5000 mg/L; COD 3000–7000 mg/L; TSS 200–1000 mg/L; and TN 300–500 mg/L. This technology can be applied in tropical and sub-tropical climates for plants. In winter, *T. angustifolia* and *C. papyrus* plants will be dormant and active again in spring and summer. However, this application can be modified by placing the plants in the greenhouse on the cover and adjusting the temperature or using geothermal energy [75].

### 3.2 Phytotoxicity test

Bioassay methods have extensively tested the toxicity of landfill leachate [18]. Toxicity tests are used to measure the number of substances in which an organism is exposed to before side effects from landfill leachate occur [133,141]. Moreover, toxicity testing using bioassays can represent chronic or acute exposure [2]. In this study, plants are used for leachate processing to be resistant to toxicity tests. The phytotoxicity test is a method that is widely used because it is fast, accurate, high sensitivity, simple, low cost, and suitable for unstable chemicals or substances [165]. Results of the plants’ ability to leachate with a content of 0%, 10%, 25%, 50%, 75%, and 100% can be seen in supplementary data.

**Fig. 2:**
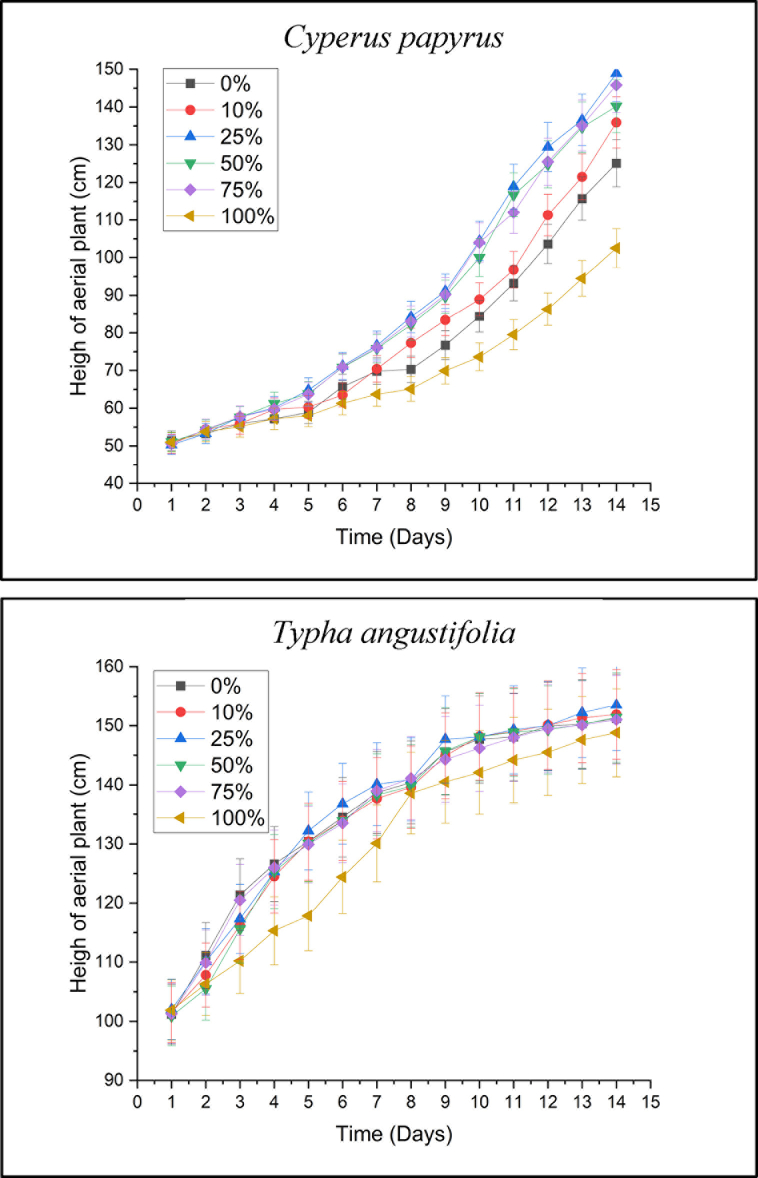
*C. papyrus* and *T. angustifolia* Growth Rate during Phytotoxicity Test.

**Fig. 3:**
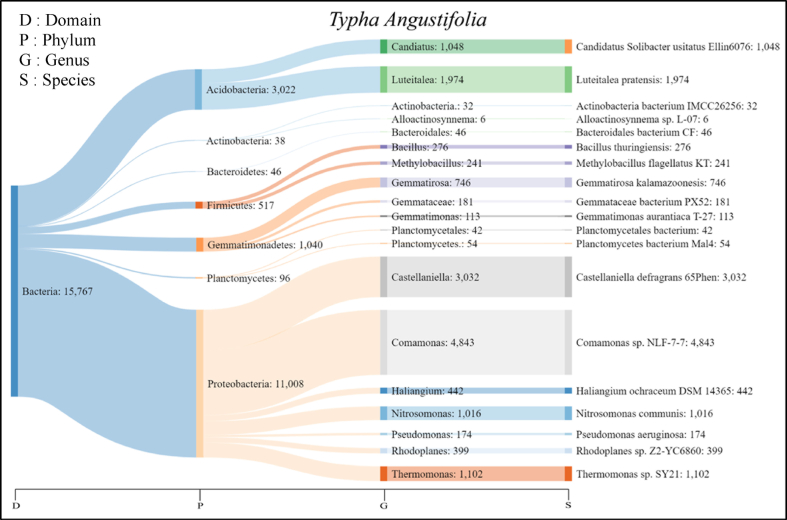
Sankey diagram of rhizobacteria leachate-resistant *T. angustifolia* showing phylum, genus, and species.

*C. papyrus* and *T. angustifolia*, were placed in the ITS Environmental Engineering Department's greenhouse, which was covered so that they will not be affected by sunlight and rain. In Fig. 3, on the first day, plants were watered with 2 L of leachate based on their respective concentrations. On the second day, there were no differences or effects occurred in all reactors. On the third day, plants were watered again because the plants needed 2 L of water every 3 days from the acclimatization results*. T. angustifolia* plants showed the fastest growth results at a leachate content of 25% > 50%> 75%. During the 14 days process, both plants did not show any withered plants. Meanwhile, leachate can provide nutrients to plants because of the presence of micro and macronutrients such as phosphate, nitrogen, Mg, Mn, Zn, Ca, and others needed by plants for growth [115,154]. Plants' growth rate during the phytotoxicity test is shown in Fig. 2.

Based on Fig. 2, plants’ growth given 100% leachate was delayed or stunted but still grew, and some shoots occurred. Another study measured the growth rate of *T. angustifolia* and showed a decrease in the higher concentrations of the chlorinated contaminant benzene [110]. Analyzed from the phase of plant death, *C. papyrus* showed a faster death than *T. angustifolia. C. papyrus* has a shorter lifespan, hence, it was more likely to turn yellow faster than other plants. Based on the research of [95]; *C. papyrus* in leachate processing with 640 mg/L of COD had an efficiency of approximately 40% which indicated that *C. papyrus* plants can accept large concentrations. Another study using the *C. indica* plant had growth inhibition along with the high leachate content but did not die, hence, the plant was tolerant of chlorophenol contaminants [42]. Leachate toxicity test on parrotfish (*Sarothodon mossambicus*) with lethal concentrations (LC-50) of 1.4% and 12% v/v at two months [13]. The result showed that both plants did not show the presence of withered plants, hence, it can be indicated that the value was 0. According to Refs. [111,161]; the high toxicity of landfill leachate was related to the mixing of organic and synthetic substances, which resulted in chemical reactions that led to the dissolution of solids into the aqueous phase. The leachate toxicity test used Tawes fish, and the results showed an LC-50 value of 0.358%, with clinical symptoms in the form of prominent eyes and brown skin [57]. A smaller EC-50 or LC-50 value indicated that the substance was increasingly dangerous for plants [10].

In addition, various leachate content may cause various effects on plant metabolism, such as inhibition of enzyme activity, mineral nutrient disturbances, water imbalance, hormonal status changes, and changes in membrane permeability. Barriers to this metabolism will be reflected in the physical changes of plants. [35]; stated that heavy metals accumulate in root cells and outside the plasma membrane. Heavy metals' effect on enzymatic activity is responsible for the metabolic processes of nutrients in roots, stems, and leaves [106]. Moreover, plants as processors must improve environmental quality without adversely affecting plants [80,122,128]. Based on the research results, the leachate concentration range of 100% will be used in the next stage of phytotoxicity studies, that is, phytodetoxification.

### 3.3 Rhizobacteria identification from *T. Angustifolia root*

Based on identification with NGS, leachate-resistant rhizobacteria can be grouped according to phylum, class, order, family, genus and species. From these results, selection was made based on the phylum which had a number >1000. In the *T. angustifolia* plant, there are 7 phyla which have 19 genera, a total of 2429 species, while the total number of individuals are 15,330. The highest number of individuals species were *Comamonas* sp. *NLF-7-7* and *Castellaniella defagrans 65Phen*. The result of rhizobacteria identification from *T. angustifolia* root is visualized with the Sankey Diagram (Fig. 3).

From the figure, the phylum Acidobacteria, Actinobacteria, Bacteroidetes, Firmicutes, Gammatimonadetes, Planctomycetes, and Proteobacteria were identified in rhizobacteria that had been given leachate. Some of the identified rhizobacteria were leachate-resistant because naturally, they were already in the leachate, which was then in the rhizosphere area. Proteobacteria is the most identified phylum. Phylum proteobacteria are often found in wastewater and wastewater treatment, which degrades pollutants [54,92,114]. Acidobacteria also has abundant individual rhizobacteria. Proteobacteria is the most abundant phylum in the sample rhizosphere and our result is consistent with previous studies [84]. Proteobacteria and Acidobacteria are ubiquitous in almost all soil types [142]. This phylum was also identified in the research of [143] in leachate processing with an Anammox-denitrification process bioreactor, which can remove various organic materials leachate.

### 3.4 Rhizobacteria identification from *C. Papyrus* root

**Fig. 4:**
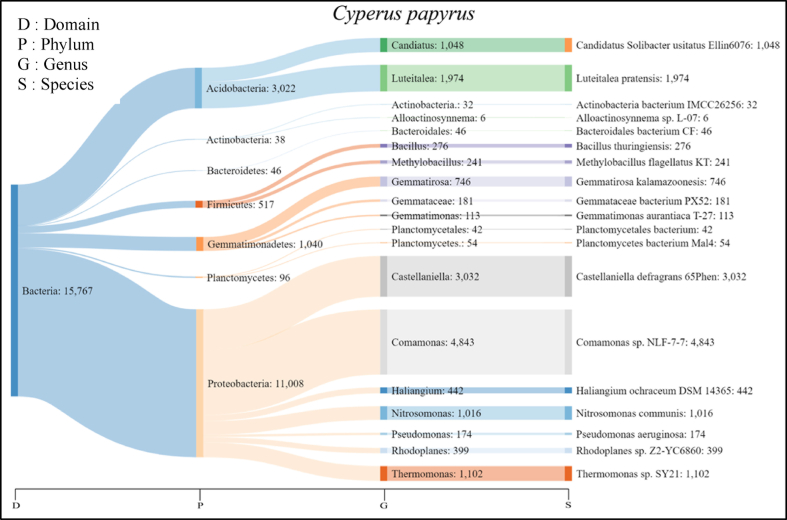
Sankey diagram of rhizobacteria leachate-resistant *C. papyrus* showing phylum, genus, and species.

From the NGS results, the number of species of rhizobacteria from *C. papyrus* root was 2386 species. The total number of individuals in *C. papyrus* was 15,766 which was more abundant than rhizobacteria in *T. angustifolia*. The highest number of individuals was *Comamonas* sp. *NLF-7-7* with 4843. The result of rhizobacteria identification from *C. papyrus* root was visualized with the Sankey Diagram (Fig. 4).

The visualization of the identification of rhizobacteria showed the association of bacteria from leachate and soil. Several general bacteria present in landfill leachate include *Bacillus, Salmonella, Citrobacter, Agrobacterium, Enterobacter, Pseudomonas, Staphylococcus,* and *Enterococcus* [37,45,83]. Naturally, bacteria in soil and plants are generally the Naturally, bacteria in soil and plants are generally the genus *Bacillus, Acetobacter, Actinobacteria,* and *Pseudomonas*, are in media with high pollutants because they can form spores and produce various enzymes [1,9,136,158]. These natural rhizobacteria live in the rhizosphere area of the *C. papyrus* plant to help detoxify leachate. Most Actinomycetes are widespread heterotrophic microorganisms and produce antibiotics and enzymes resistant to certain stress factors such as heavy metals. Actinomycetes and Bacillus are also known to produce amylase, xylanase, protease, lipase, cellulase, and others. This enzyme plays a role in degrading organic matter [5,66].

### 3.5 Analysis of rhizobacterial diversity

Next Generation Sequencing (NGS) identified the phylum in leachate-resistant rhizobacteria were 7, with 19 dominant species. The potential of the 19 dominant species can assist leachate processing by degradation of organic matter and reduction of inorganic matter in leachate (Table 2).

**Table 2:** Diversity of phylum, genus, and species of leachate-resistant rhizobacteria in *C. papyrus* and *T. angustifolia* based on NGS.

Rhizobacteria			Number of individuals			
Phylum	Genus	Dominant species	*T. angustifolia*	*C. papyrus*	Potency	Reference
Proteobacteria	*Comamonas*	*Comamonas* sp. *NLF-7-7*	2097	4843	The denitrification process plays a role in reducing nitrate and nitrogen	[107]
	*Pseudomonas*	*Pseudomonas aeruginosa*	63	174	Degradation of various organic compounds and heavy metal in leachate	[86,87]
	*Rhodoplanes*	*Rhodoplanes* sp. *Z2-YC6860*	1095	399	Potential in the degradation of phenol, cyanide, benzoate and naphthalene	[38,132]
	*Castellaniella*	*Castellaniella defragrans 65 Phen*	2490	3032	Tolerant of low pH and plays a role in the denitrification process	[22]
	*Nitrosomonas*	*Nitrosomonas communis*	1102	1016	Can oxidize ammonia	[118]
	*Haliangium*	*Haliangium ochraceum DSM 14365*	1776	442	Has the ability to remove Total Nitrogen	[4]
	*Thermomonas*	*Thermomonas* sp. *SY21*	442	1102	Convert H _2_ + CO _2_ to CH _4_	[137].
Firmicutes	*Bacillus*	*Bacillus thuringiensis*	332	276	Bacteria decompose pathogens in leachate and have the ability to remove COD up to 90%, NH_3_->50%, and Humic Acid>40%.	[127,139]
	*Methylobacillus*	*Methylobacillus flagelatus KT*	239	241		
Bacteroidetes	*Bacteroidales*	*Bacteroidales bacterium CF*	32	46	*Bacteroidetes* convert Acetate, propionate, H_2_, and CO_2_ into the main end products of fermentation	[21]
Acidobacteria	*Candidatus*	*Candidatus Solibacter usitatus Ellin6076*	1563	1048	Can break down, utilize and biosynthesis of polysaccharides and is resistant to temperature fluctuations	[59]
	*Luteitalea*	*Luteitalea pratensis*	1820	1974		
Actinobacteria	*Actinobacteria*	*Actinobacteria bacterium IMCC26256*	70	32	Degradation of organic and inorganic materials such as heavy metals	[12]
	*Alloactinosynnema*	*Alloactinosynnema* sp. *L-07.*	6	6		
Gemmatimonadetes	*Gemmatirosa*	*Gemmatirosa kalamazoonesis*	1588	746	Phenol degradation	[90]
	*Gemmatimonas*	*Gemmatimonas aurantiaca T-27*	223	113		
	*Gammataceae*	*Gemmataceae bacterium PX52*	201	181		
Planctomycetes	*Planctomycetales*	*Planctomycetales bacterium*	97	42	Degradation of organic matter through hydrolysis and carbonylation	[148,149]

As shown in the table, there are two phyla of leachate-resistant rhizobacteria that have individuals in *C. papyrus* and *T. angustifolia* >1,000, namely Proteobacteria and Acidobacteria. Species in the phylum Proteobacteria, namely *Comamonas* sp. *NLF-7-7, Castellaniella defragrans 65 Phen*, and *N. communis*, while in the phylum Acidobacteria is *Candidatus Solibacter usitatus Ellin6076* and *Luteitalea pratensis*. Acidobacteria Species of rhizobacteria in plants can help with leachate processing and help plant growth [14]. The genus *Comamonas* has an excellent ability to reduce nitrate in the denitrification process by combining the oxidation of the substrate present in the leachate with the reduction of nitrate, thus playing an important role in the elimination of nitrate and organic compounds [130,150]. *Comamonas* has a strong aggregation ability, thus resulting in accelerated phenol degradation [87].

*Castellaniella* is found in sludge at leachate treatment plants as denitrifying bacteria [163]. In addition, *Castellaniella* can reduce nitrate to nitrite and nitrite to nitrogen gas [77]. Moreover, *Castellaniella* uses substrates such as d-arabinose, d-glucose, d-galactose, cellobiose, l-fucose, acetate, dl-3-hydroxybutyrate, valerate, fumarate, malate, succinate, and formate [73]. The genus *Nitrosomonas* is primarily responsible for ammonium oxidation in leachate treatment [76,153]. Furthermore, *Nitrosomonas* is dominantly observed in partial nitrification-based wastewater treatment systems, such as urban wastewater, aquaculture wastewater, and landfill leachate [76,153]. The leachate treatment can take place if the presence of these bacteria is assisted by sufficient oxygen to ensure ammonium oxidation [56]. Meanwhile, *Candidatus* can oxidize ammonium with nitrite as an electron acceptor under anaerobic conditions [63]. The ecological relationships of *Candidatus* and other genera in the association network were a form of mutualism to degrade pollutants [55,152]. Some microorganisms can use organic acids as electron donors [99,112,113]. *Luteitalea* produces dissimilatory nitrite reductase (nrfHA), which catalyzes nitrite to ammonia [41,62].

Although the degradation time can be increased, naturally, leachate bacteria can degrade pollutants in the long term. One of these processing mechanisms is the role of rhizobacteria carried out in this study. Afterward, rhizobacteria were identified as having important roles in leachate processing and can optimize processing.

### 3.6 Phytodetoxification comparison

#### 3.6.1 Single plant uses versus combination

**Fig. 5:**
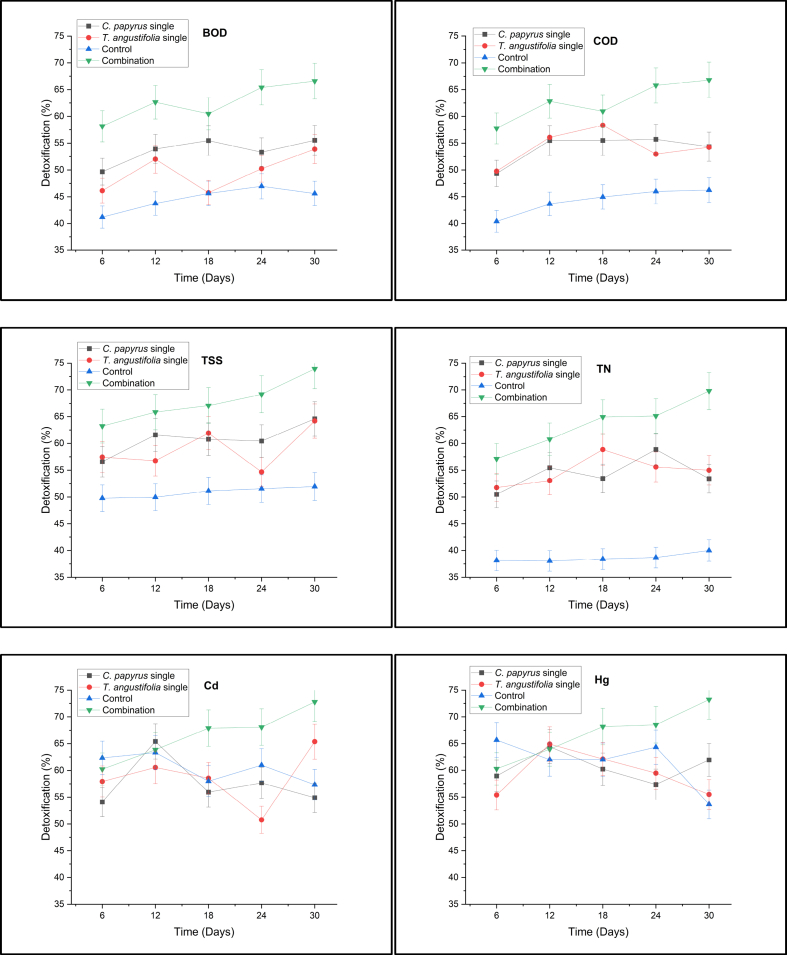
Phytodetoxification of each parameter.

Plants can receive native leachate in high concentrations. Then, the detoxification ability of plants was investigated, and found that the detoxification value of pollutants in leachate was up to >50%. The graph of the percentage of detoxification for COD and BOD can be seen in Fig. 5.

Based on the results of statistical analysis, the concentrations of BOD, COD, TN, TSS, Cd, and Hg of plants after treatment were the same at different times; on the contrary, the use of different plants provides significant differences (p < 0.05) in terms of different subsets of *T. angustifolia, C. papyrus*, and combination of plants. The type of plant showed the highest F value, thereby indicating that it was an influential factor in leachate processing because different plants had different processing capacities.

Decreasing the BOD_5_/COD ratio can affect the COD by lowering its concentration. On the one hand, [40]; stated that besides plants, plant media turn out to be the next major role in COD removal. On the other hand, bacterial activity may also be the cause of COD reduction in constructed wetlands [48,102,159]. The media can provide a suitable surface on which a biofilm can form while adhering bacteria may aid in COD removal [93,140]. One of the typical leachate characteristics is it has an extremely low BOD_5_/COD ratio (<0.1), which can be said that most of the organic compounds in leachate are non-biodegradable [17,74,123] The low percentage of COD removal during operation is possible because of the higher concentration of non-biodegradable organic compounds in the leachate [24,39,65]. The initial leachate BOD/COD value is 0.3–0.4, hence, it is classified as biodegradable. The leachate treatment performance of the system increased from biodegradable to stable leachate, namely BOD/COD>0.9 [31,120], and the possibility of processing assistance from microbial films on the media [119]. TSS is another parameter that is in the Regulation of the Minister of Environment and Forestry, Indonesia No. 59 of 2016 about Leachate Quality Standards.

In addition, the TSS concentration significantly decreased in the process with the lower residence time. However, the difference between the TSS concentrations became insignificant as the residence time increased. This observation is related to solids remobilization in constructed wetlands with longer residence times. [64]; noted that when solids remobilization occurs, it limits the effectiveness of wetland systems with longer residence times. Meanwhile, TSS was greatly reduced by media in constructed wetlands as the plant was decreasing another parameter, it occurs because constructed wetland media has a mixture of materials that potentially filtered TSS [94]. TN is another parameter that is included in the quality standard.

The detoxification in the three plants is around 50%. Reduction of TN from landfill leachate is very important because excessive levels of TN cause serious water quality problems. In addition, there are some mechanisms and options for TN removal processes in wetland systems. These possible options include plant uptake, evaporation, sedimentation, nitrification, and denitrification [49,124,157]. However, high porosity from wetland media enhances the TN removal from water through its high ion exchange capacity. The higher porosity may have increased the growth of microorganisms, thereby resulting in higher ammonia removal [108].

The presence of competitive ions such as heavy metals in the leachate resulting the adsorption capacity being way lesser [96]. Nutrient absorption by plants and microbial activity in wetlands is directly or indirectly affected by temperature. During the study, the average temperature in September is 29 °C. Nitrification is often thought to be temperature-dependent in constructed wetlands. It was reported that the nitrification rate in constructed wetlands became difficult at 10 °C [91]. Microorganism-mediated oxidation of organic matter and nitrogen transformation is also affected by temperature (i.e., climatic conditions). The effluent TN concentration higher than the influent value indicates the occurrence of nitrification. Oxygen is used for nitrification and organic removal. The oxygen produced by photosynthesis during the day also supports oxygen requirements for organic stabilization and nitrification. Meanwhile, in constructed wetland systems, denitrification depends on the presence of nitrogen nitrate and organic carbon. Environmental factors such as pH, temperature, microbial attachment surface area, and dissolved oxygen concentration also affect denitrification [129].

**Fig. 6:**
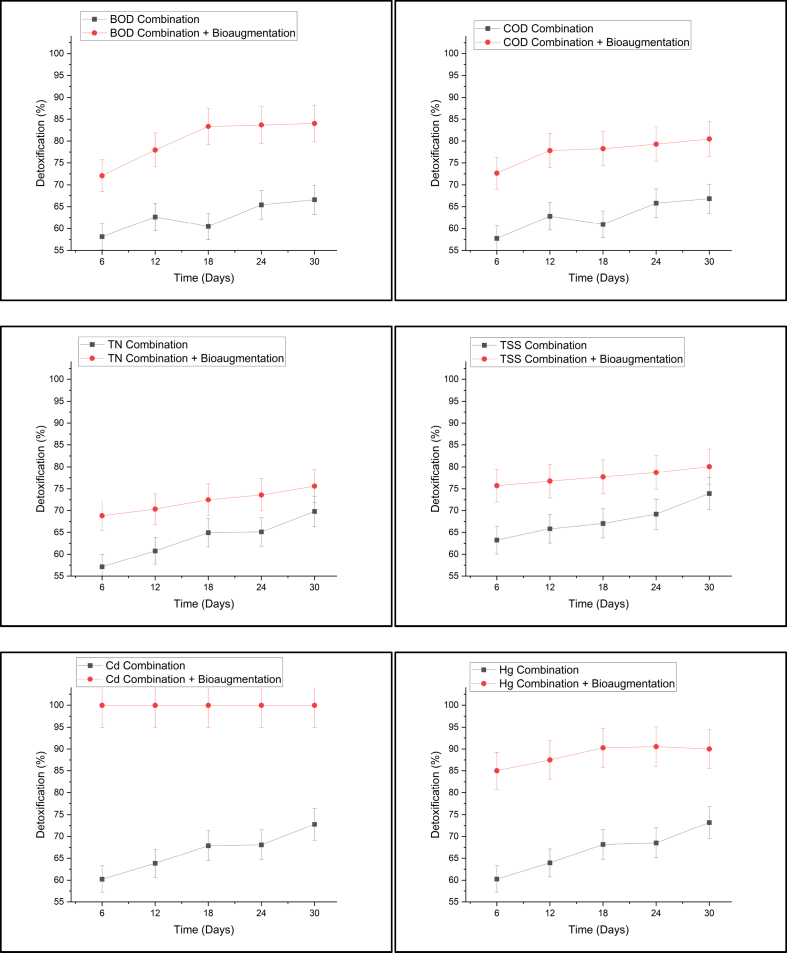
Presentation of phytodetoxification without bioaugmentation versus bioaugmentation.

Leachate contains organic and inorganic materials such as heavy metals. Heavy metal parameters that are in the leachate quality standard are Cd and Hg. The concentration of Cd in the leachate of the Griyomulyo landfill fluctuated and the sampling met the quality standard several times. The results of Cd detoxification in Fig. 6 show high efficiency and in all data, the Cd concentration is far below the quality standard until it is not identified. According to Ref. [98]; plants have substances that are excreted by plant roots in constructed wetlands for the microbial activity of tannic acid, gallic acid, and other compounds. The microbial activity produces phytometallophores (phytosiderophores) which are non-proteinogenic amino acid exudates that chelate and mobilize heavy metals [29]. Then, the chelated metal can be taken up by roots and subsequently transported to the above-ground plant parts [151].

Heavy metals are present in landfills as by-products of various consumer products such as batteries, plastics, ceramics, and electronics. Landfill leachate has a very low Hg value and is close to the quality standard value. The results of Hg detoxification have an efficiency of around 60%, hence, they can meet quality standards. The main mechanism in the transfer of heavy metals in constructed wetlands is through biological pathways and chemical precipitation along with the binding of organic matter, absorption to the soil surface and plant roots, and filtration of suspended solids by the root system and soil [44,109]. In addition, plants can influence the biogeochemistry of constructed wetlands which can alter metal retention [138]. Thus, the contribution of each of these processes to the total metal removal is highly dependent on environmental conditions, particularly pH and oxidation-reduction as well as the nature of the metal itself.

#### 3.6.2 Bioaugmentation versus without bioaugmentation

The identification results obtained potential bacteria in assisting leachate processing, potential bacteria were obtained from the number, living medium, ease of adaptation, and were known to be leachate-resistant. The ratio between the types of bacteria was the same. Several additional bacteria have been obtained from the research which showed that 2 (v/v) soil media showed best processing results [103]. Results of leachate detoxification with the addition of bacteria for all parameters are shown in Fig. 6.

Fig. 6 shows that the percentage of TN detoxification with the addition of bacteria was about 20% higher than without bioaugmentation. This is following the research conducted which used *Bacillus* sp. For leachate treatment and has 40% greater ability than without the addition of bacteria [33]. Nitrification is a natural water purification process by oxidizing potentially toxic ammonia to non-toxic nitrate, and bacteria Nitrosomonas and Nitrobacter play important roles in this process [60]. Inoculation of nitrifying bacteria can reduce the organic load in the water [145]. The study used a mixture of bacteria containing *Nitrosomonas* sp.*, Nitrosococcus* sp.*, Nitrobacter* sp.*, Bacillus* sp.*, Aerobacter* sp., and *Pseudomonas* sp. Can reduce 68% organic matter load within 4 days [145].

In addition to having a high ability to detoxify organic matter, inorganic materials such as heavy metals can also be reduced. *Pseudomonas* sp. is Cd^2+^ and Cu^2+^-resistant bacteria that synthesize proteins that are induced and then binds those cations as metal accumulation on the outer membrane [51,88]. The extracellular barrier is the shield of microorganisms that prevent heavy metals from entering cells. Thus, cell wall and plasma membrane play important roles during this stage in inhibiting heavy metals from entering the cell. Microorganisms can naturally produce extracellular polysaccharides (EPSs) which are found on the outer surface to support the absorption of metal ions and prevent them from penetrating the cell surface. Some bacteria, such as *P. aeruginosa, P. stutzeri, Arthrobacter* sp.*,* and *Rhizobium metallidurans*, show the ability to bind metals extracellularly [53].

## 4 Conclusion

*C. papyrus* and *T. angustifolia* species can receive up to 100% leachate from exposure for 14 days through a range-finding test and qualitative and quantitative physical observations. The highest number of rhizobacterial species found is potential in leachate processing. The bacteria that were added to the reactor were *P. aeruginosa, B. cereus*, and *N. communis*. The combination of plants and bioaugmentation has higher detoxification efficiency than without bioaugmentation. The highest detoxification results were obtained at the reactor that bioaugmented with rhizobacterial for the parameters COD, BOD, TSS, TN, Cd, and Hg are more than 70%.

## Author contribution statement

Isni Arliyani: Conceived and designed the experiments; Performed the experiments; Analyzed and interpreted the data; Contributed reagents, materials, analysis tools or data; Wrote the paper.

Bieby Voijant Tangahu; Sarwoko Mangkoedihardjo: Conceived and designed the experiments; Analyzed and interpreted the data; Wrote the paper.

Enny Zulaika; Setyo Budi Kurniawan: Analyzed and interpreted the data; Wrote the paper.

## Funding statement

This work was supported by the 10.13039/100009950Ministry of Education, Culture, Research, and Technology, Indonesia through the PMDSU program.

## Data availability statement

Data included in article/supp. Material/referenced in article.

## Declaration of competing interest

The authors declare that they have no competing interest to influence the work reported in this paper.
