# Supplementary figures and images for: Enhanced leachate phytodetoxification test combined with plants and rhizobacteria bioaugmentation
Source: Heliyon. 2023 Jan 13;9(1):e12921. doi: 10.1016/j.heliyon.2023.e12921 (PMC9938419; doi:10.1016/j.heliyon.2023.e12921)

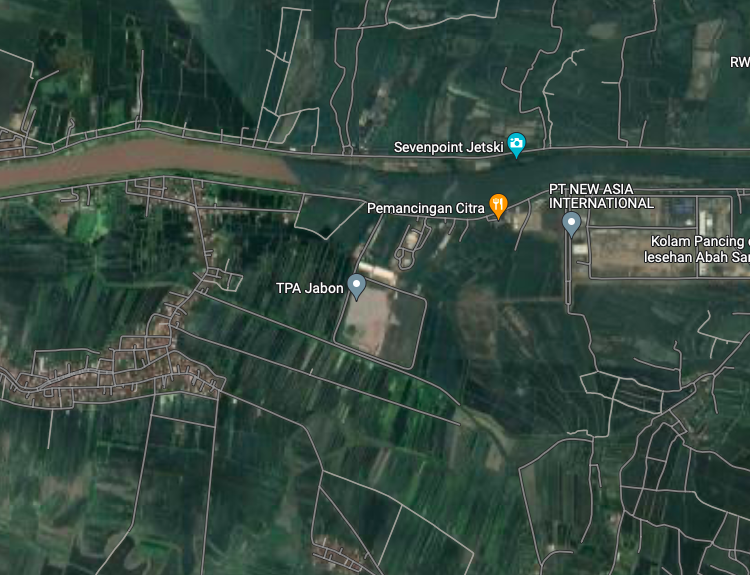


Griyomulyo landfill

184°S7°32'53"S 112°45'50" E

**Figure 1.** Sampling Location

Day 0 Day 14

**Figure 2.** Range Finding Test Results

Supplement: Multimedia component 1 [file mmc1.docx]
